# Complex signatures of genomic variation of two non-model marine species in a homogeneous environment

**DOI:** 10.1186/s12864-018-4721-y

**Published:** 2018-05-09

**Authors:** Erica S. Nielsen, Romina Henriques, Robert J. Toonen, Ingrid S. S. Knapp, Baocheng Guo, Sophie von der Heyden

**Affiliations:** 1 Evolutionary Genomics Group, Department of Botany and Zoology, University of Stellenbosch, Private Bag X1, Matieland,, 7602 South Africa; 2 Hawaiʻi Institute of Marine Biology, School of Ocean and Earth Science and Technology, University of Hawaiʻi at Mānoa, Kāneʻohe, HI 96744 USA; 3 The Key Laboratory of Zoological Systematics and Evolution, Institute of Zoology Chinese Academy of Sciences, Beijing, 100101 China

**Keywords:** Genomics, RAD-seq, Comparative phylogeography, SNP, Local adaptation, Population differentiation

## Abstract

**Background:**

Genomic tools are increasingly being used on non-model organisms to provide insights into population structure and variability, including signals of selection. However, most studies are carried out in regions with distinct environmental gradients or across large geographical areas, in which local adaptation is expected to occur. Therefore, the focus of this study is to characterize genomic variation and selective signals over short geographic areas within a largely homogeneous region. To assess adaptive signals between microhabitats within the rocky shore, we compared genomic variation between the Cape urchin (*Parechinus angulosus*), which is a low to mid-shore species, and the Granular limpet (*Scutellastra granularis*), a high shore specialist.

**Results:**

Using pooled restriction site associated DNA (RAD) sequencing, we described patterns of genomic variation and identified outlier loci in both species. We found relatively low numbers of outlier SNPs within each species, and identified outlier genes associated with different selective pressures than those previously identified in studies conducted over larger environmental gradients. The number of population-specific outlier loci differed between species, likely owing to differential selective pressures within the intertidal environment. Interestingly, the outlier loci were highly differentiated within the two northernmost populations for both species, suggesting that unique evolutionary forces are acting on marine invertebrates within this region.

**Conclusions:**

Our study provides a background for comparative genomic studies focused on non-model species, as well as a baseline for the adaptive potential of marine invertebrates along the South African west coast. We also discuss the caveats associated with Pool-seq and potential biases of sequencing coverage on downstream genomic metrics. The findings provide evidence of species-specific selective pressures within a homogeneous environment, and suggest that selective forces acting on small scales are just as crucial to acknowledge as those acting on larger scales. As a whole, our findings imply that future population genomic studies should expand from focusing on model organisms and/or studying heterogeneous regions to better understand the evolutionary processes shaping current and future biodiversity patterns, particularly when used in a comparative phylogeographic context.

**Electronic supplementary material:**

The online version of this article (10.1186/s12864-018-4721-y) contains supplementary material, which is available to authorized users.

## Background

Disentangling the contributions of evolutionary processes through space and time is central to interpreting genetic signals of population dynamics and understanding how the environment shapes a species’ distribution [1, 2]. The evolutionary trajectories of species are also important for conservation management, particularly under anthropogenically driven environmental change, which has heavily influenced the spatial distribution of many species over relatively short evolutionary timescales [3]. From a conservation perspective, intraspecific genomic variation is a principal component of evolutionary diversification, and is an important feature to help prioritize populations with higher adaptive potential [4–7].

An increasing number of studies are utilizing high-throughput sequencing methodologies to assess the intraspecific adaptive potential of species and evaluate how genetic variation is associated with environmental heterogeneity [8–10]. However, the majority of studies directed at identifying genes under selection do so with model organisms, and over large areas with strong environmental gradients, where local adaptation is to be expected (see for example [11–16]). Fewer studies characterize genetic differentiation over relatively small and/or environmentally homogeneous regions (although see [17, 18] for microhabitat examples), leaving genome-wide variation of species within these types of environments unexplored. Furthermore, studies utilizing genomic data to conduct comparative phylogeographic analyses remain underrepresented in the literature, although the power of including multiple taxonomic groups into evolutionary studies is well recognized for mitochondrial DNA (mtDNA), nuclear DNA and microsatellites [19–21]. Despite the marked increase in data available with genomic tools, comparative analyses are still required for understanding the underlying processes shaping genomic variation across landscapes, as well as producing representative conservation plans [22, 23]. Comparative approaches also provide opportunities to test whether different species respond to the same environmental drivers in similar ways, or whether signals of selection differ across species and their populations [24, 25], and the scales at which selection acts [17].

**Fig. 1:**
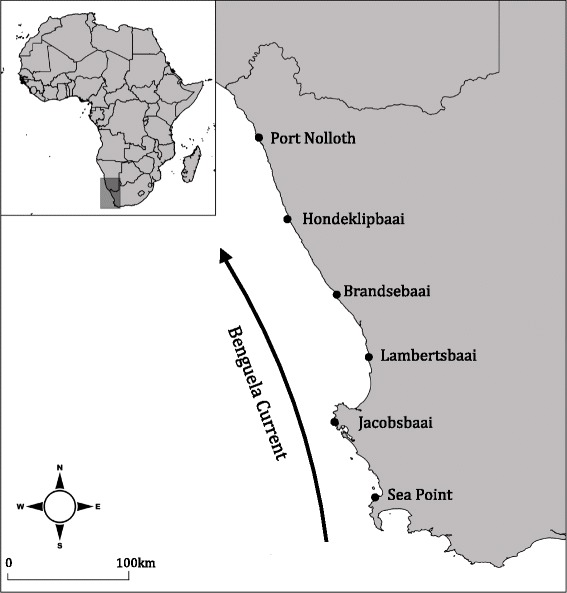
The six sample locations in which 40 individuals of each species were collected for genomic analyses, along with the dominant current in the study region

The South African west coast is a relatively short, linear and homogeneous coastline with little variation in sea surface temperature (SST) and primary productivity (Fig. 1; [26]). The west coast is a highly-threatened region of the South African coastline, with exposure to diamond, oil and gas mining as well as fishing pressures [27, 28]. It is situated within the southern Benguela Upwelling System, one of the most productive eastern boundary currents in the world [29], and is heavily influenced by the Benguela Current, which flows along the South African coastline from south to north (Fig. 1; [30, 31]). Despite this dominant northward flowing current, multiple studies show variable genetic structuring for species along the South African west coast [32–37], with evidence of local oceanography such as eddies appearing to shape genetic differentiation of coastal species in this region [37]. We chose six rocky shore sample sites within the study region, which are evenly spaced at ~ every 100 km of the coastline (Fig. 1). These sample sites were chosen to capture the full range of coastal habitat types, habitat conditions, ecoregions and protection levels along the South African west coast [27, 28].

Despite geographic conditions often playing important roles in shaping the biodiversity patterns of species [38, 39] the effects of environmental and ecological features on intraspecific genomic variation and adaptation still remain unclear for many sessile marine species with planktonic larvae [40]. To investigate the phylogeographic patterns of these marine species with larval distribution stages, we selected two rocky shore study species, namely the Cape urchin (*Parechinus angulosus,* Leske 1778) and the Granular limpet (*Scutellastra granularis*, Linneaus 1758), collecting 40 individuals from each sample site. We chose these two taxa as they represent different ecological niches, with the Granular limpet being a high shore species with a relatively short pelagic larval duration (PLD; ~ 10 days) and the Cape urchin being a mid to low shore species with a relatively long PLD (~ 50 days [41, 42]). Although they have similar range distributions, previous phylogeographic patterns measured with mtDNA Cytochrome Oxidase subunit 1 (*COI*) showed contrasting genetic structuring for the two species, with the limpet displaying low genetic differentiation, compared to the high levels of genetic differentiation of the urchin [35, 42]). Because non-model species (i.e. those without annotated genomes) are underrepresented in genomic studies, and because of the lack of genome projects focused on South African marine species, we chose to study two non-model organisms and utilize de novo assemblies as reference sequences [43–45].

Here we use pooled ezRAD sequencing, a size-selection-based reduced representation genomic sequencing approach [46], to build on previous comparative studies using mtDNA markers [32, 33, 35], which should provide more powerful results for genome-wide variation and selective signals on two non-model species. We also use genome-wide SNP datasets to compare patterns of genomic variation and population structure between species. We expect to find high levels of genomic diversity, yet low levels of selection in both species, and to identify genes associated with different selective pressures than those previously identified in marine taxa occurring in regions with larger environmental gradients. Largely, this study aims to compare the distribution of genomic variation between two sessile marine species, so as to better understand the processes shaping the evolutionary history of species within a highly productive and threatened coastline.

## Results

### Sequencing and assembly

A total of 35.4 million paired reads were obtained from the Granular limpet libraries, with an average of 5.9 million paired reads per sample (from hereon referred to as population). The de novo assembly produced a total of 452,948 contig sequences, which were combined to create the reference sequences for all downstream analyses (Additional file 1: Table S1). The Granular limpet de novo assembly was roughly 180 Mb in length, the longest contig was 12,107 bp, and the N50 and L50 were 717 bp and 87,790 bp, respectively (Additional file 1: Table S1). A total of 25 million reads were mapped onto the reference sequences, and the number of mapped reads ranged from 3.4 to 4.7 million per population (Table 1). The average length of mapped reads for the limpet was 252 bp, and the average base quality of the mapped reads was 35.3 Phred.

**Table 1:** The sample site, number of mapped reads (# of mapped reads)*,* number of SNPs calculated by PoPoolation (# of SNPs), and genetic diversity indices (Tajima’s π and Watterson’s *θ*_*W*_) are shown for the limpet, *S. granularis* and the urchin, *P. angulosus*. The number (#) and percentage (%) of private SNPs are also shown per population for both study species

North to south orientation	Sample site	# of mapped reads	# of SNPs	Tajima’s *π*	Watterson’s *θ*_*W*_	# of private SNPs	% of SNPs that are private
*S. granularis*							
North	PN	3,372,943	49,455	0.009	0.010	15,496	0.313
	HB	4,263,248	113,678	0.011	0.012	36,035	0.317
	BB	4,756,683	151,071	0.011	0.012	54,657	0.362
	LB	3,674,168	91,767	0.010	0.011	18,957	0.207
	JB	4,613,158	152,423	0.012	0.013	72,096	0.472
South	SP	4,348,563	135,499	0.011	0.012	57,630	0.425
*P. angulosus*							
North	PN	3,309,914	100,849	0.011	0.012	62,007	0.615
	HB	2,304,239	18,682	0.006	0.007	6961	0.373
	BB	3,234,311	72,024	0.009	0.010	18,735	0.260
	LB	3,133,465	69,921	0.009	0.010	25,390	0.363
	JB	4,436,171	98,110	0.009	0.011	34,826	0.355
South	SP	2,423,381	24,747	0.007	0.008	8905	0.360

Sample site abbreviations are as follows: *SP* Sea Point, *JB* Jacobsbaai, *LB* Lambertsbaai, *BB* Brandsebaai, *HB* Hondeklipbaai, *PN* Port Nolloth

The Cape urchin libraries yielded 27.3 million paired reads, with an average of 4.5 million paired reads per population, resulting in 453,847contig sequences from the de novo assembly (Additional file 1: Table S1). This assembly was ~ 200 Mb in length, the longest contig was 265,371 bp, and the N50 and L50 were 719 and 94,187, respectively (Additional file 1: Table S1). After mapping, 19 million reads were aligned to the urchin reference sequences, and total mapped reads ranged from 2.3 to 4.4 million per population (Table 1). The average length of mapped reads for the Cape urchin samples was 229 bp, and the average base quality of the mapped reads was 35.1 Phred.

### Genome-wide variation

The number of single nucleotide polymorphisms (SNPs) identified by PoPoolation v1.2.2 [47] varied among Granular limpet populations, with Port Nolloth having the lowest number of SNPs with 49,455 and Jacobsbaai having the highest with 152,423 (Table 1). The within-population average nucleotide diversity of the Granular limpet ranged from 0.009 to 0.012 for Tajima’s *π* and 0.010 to 0.013 for Watterson’s *θ*_*W*_ (Table 1). A total of 55,409 SNPs were identified with PoPoolation2 v1.201 [48] across all limpet populations, Port Nolloth again had the lowest number of SNPs, and Brandsebaai had the greatest (Table 2). As Popoolation2 is not capable of calculating diversity indices (i.e. Tajima’s *π* and Watterson’s *θ*_*W*_), we calculated total heterozygosity from the GenePop files used in the outlier detection analyses. The total heterozygosity was highly similar between limpet populations, ranging between 0.082 to 0.084 (Table 2). The number of private SNPs (SNPs unique to certain locations) within the limpet populations ranged from 9 to 226, and the percentage of population-specific private SNPs ranged between 0.017% to 0.421% (Table 2).

**Table 2:** The sample site, number of SNPs identified from all populations combined in Popoolation2 (# of total SNPs), and the total heterozygosity (Ht) are shown for *S. granularis* and *P. angulosus*. The number (#) and percentage (%) of those SNPs that were private are also shown for both species. Population abbreviations are provided in Table 1

North to south orientation	Sample site	# of mapped reads	# of total SNPs	Ht	# of private SNPs	% of SNPs that are private
*S. granularis*						
North	PN	3,372,943	47,090	0.082	35	.074
	HB	4,263,248	53,686	0.084	226	.421
	BB	4,756,683	54,862	0.084	15	.027
	LB	3,674,168	52,352	0.084	9	.017
	JB	4,613,158	53,238	0.084	29	.054
South	SP	4,348,563	53,003	0.082	42	.079
*P. angulosus*						
North	PN	3,309,914	6665	0.058	14	.210
	HB	2,304,239	5204	0.052	2	.038
	BB	3,234,311	7775	0.054	2	.026
	LB	3,133,465	7633	0.054	6	.078
	JB	4,436,171	7404	0.054	5	.068
South	SP	2,423,381	5625	0.057	2	.036

The Cape urchin populations showed greater variation in the number of SNPs identified by PoPoolation, with the lowest (24,747) for Sea Point and the highest (100,849) for Port Nolloth (Table 1). The population-specific nucleotide diversity values, Tajima’s *π* and Watterson’s *θ*_*W*_, ranged from 0.006 to 0.011 and 0.007 to 0.012, respectively (Table 1). The more stringent criteria used in PoPoolation2 identified a total of 8,386 SNPs, and the within population number of SNPs ranged from 5,204 to 7,775 (Table 2). The number of private SNPs ranged from two to 14 SNPs, and the percentage of private SNPs ranged from 0.026% to 0.21% (Table 2). The total heterozygosity varied more between the Cape urchin populations compared to the limpet, with values ranging from 0.052 to 0.057 (Table 2). The Cape urchin showed considerably lower levels of population-specific diversity, with average heterozygosity being 0.055 and 0.083 respectively (Table 2).

The allele frequency spectrum plots showed minor differences in allele frequencies between populations when calculated from SNPs identified in Popoolation, and highly similar frequencies between populations when calculated from Popoolation2 SNPs (Additional file 1: Figure S1-S4).

### Detection of outlier loci

A total of 55,409 SNPs from the Granular limpet populations were included in the outlier detection analyses. Bayescan analyses identified 98 outlier loci within the limpet populations, all of which identified as under diversifying selection. PCAdapt [49] selected a larger amount of outlier loci compared to Bayescan, with a total of 355 outliers. Only 34 outlier SNPs were detected by both Bayescan and PCAdapt, and the number of outliers within each population ranged from 14 to 30 (Table 3). Hondeklipbaai was the only location to have private outlier SNPs, with nine unique outlier loci. The 34 outliers chosen by both Bayescan and PCAdapt were located on 17 contigs.

**Table 3:** The number of outlier SNPs identified in each limpet (*S. granularis*) and urchin (*P. angulosus*) population. Population abbreviations are provided in Table 1

North to south orientation	Sample site	Number of outlier SNPs	
		*S. granularis*	*P. angulosus*
North	PN	14	7
	HB	30	5
	BB	16	8
	LB	15	8
	JB	16	8
South	SP	19	8
	Shared by all sites	8	4

Of the 17 contigs that were BLASTed, 76% of them successfully paired with sequences with an E-value of 10^− 5^ or above. All matches were with hypothetical proteins from the Owl limpet, *Lottia gigantea,* genome and most were matched to conserved protein domains such as histone, homeodomain and ribonuclease H-like domains. (Additional file 1: Table S2). When outlier contigs were mapped to the *L. gigantea* genome to identify neighboring genes, the only non-hypothetical protein match was to the pol-like protein.

Of the 8,386 Cape urchin SNPs analyzed, 12 were selected as outlier loci by Bayescan, all of which were identified as under balancing selection. The PCAdapt outlier analysis identified a total of 61 outlier loci. Eight outlier loci were identified by both methods, with half of these outlier loci shared across all populations. Within the remaining half of outlier loci, three were found in all populations except in Hondeklipbaai, and one was found in all populations except for Port Nolloth. The eight outlier loci were located on seven contigs. Of the seven outlier contigs, four had BLAST results with significant E-values, and matched with predicted proteins from the Purple urchin, *Strongylocentrotus purpuratus,* genome (Additional file 1: Table S2). The respective domains of the predicted proteins included the histone H3, retroelements and mobile elements, and the Endonuclease/Exonuclease/Phosphatase family (Additional file 1: Table S2). When the four outlier contigs were mapped onto the *S. purpuratus* genome to identify neighboring genes, the only identified gene was the cysteine-rich motor neuron 1 protein precursor.

### Population genomic structuring

The average pairwise *F*_ST_ values across all SNPs were similar between the two species. The values for the Cape urchin ranged from 0.006 to 0.019, and the values for the Granular limpet ranged from 0.008 to 0.013 (Additional file 1: Tables S3 & S4). The Cape urchin had a larger range of *F*_ST_ values per locus, with a minimum *F*_ST_ of 2.1e^− 5^ and a maximum *F*_ST_ of 0.951, compared to the minimum and maximum per locus *F*_ST_ values of 2.3e^− 5^ and 0.785 for the Granular limpet*.*

**Fig. 2:**
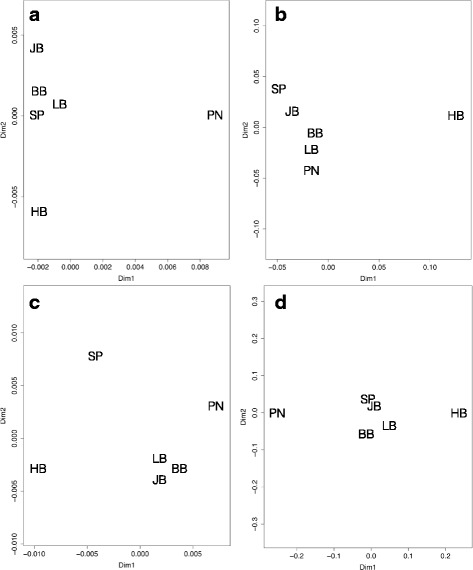
Genetic differentiation displayed in PCoA plots, calculated from non-outlier SNPs (**a**, **c**) and outlier SNPs (**b**, **d**) for the limpet, *S. granularis* (**a, b**) and the urchin, *P. angulosus* (**c**, **d**) populations. Population abbreviations are provided in Table 1

To assess population genomic structuring, we first removed the outlier SNPs to calculate ‘neutral’ pairwise *F*_*ST*_ values. We subsequently calculated ‘outlier’ *F*_*ST*_ values using only the outlier SNPs. The genomic differentiation patterns based on *F*_ST_ values from the neutral SNPS differed from those based on outlier SNPs for both species (Fig. 2). Interestingly, the populations within the mid-coast (i.e. Jacobsbaai, Lambertsbaai, and Brandsebaai) tended to cluster together for both species, for both non-outlier and outlier loci (Fig. 2). Furthermore, the population of Hondeklipbaai was genomically distinct in both the neutral and outlier analyses for both study species (Fig. 2).

Both species showed no signals of isolation-by-distance (IBD), based on the full SNP datasets, as well as neutral and outlier SNP datasets (Additional file 1: Table S5). The K-means clustering analyses with fastStructure v1.0 [50] resulted in wide ranges of K values for the neutral and outlier datasets for both species and the admixture plots did not display strong signals of structure (data not shown).

## Discussion

The South African west coast harbors highly productive coastal communities [41, 50], but is also widely impacted by anthropogenic development [27]. It is thus vital to understand the genomic patterns and adaptive potential of marine organisms inhabiting this region, because even basic population genetic metrics have been shown to play important roles in conservation planning [42, 51, 52]. The region is also of interest as, compared to other studies that have identified genome-wide variation [14–16, 53–55], it does not experience strong environmental heterogeneity, and so all else being equal, populations within this area might be expected to have fewer signals of local adaptation [56, 57].

Using a high-throughput sequencing approach, we constructed SNP datasets and identified loci that appear to be under selection for two non-model rocky shore species within this region. In line with our predictions, we found relatively low numbers of outlier SNPs within each species, and identified outlier genes associated with different selective pressures than those previously identified in marine taxa occurring in regions with larger environmental gradients [11–16]. We also found differences in outlier SNP patterns between the two species (Fig. 2), possibly due to different selective forces acting on high and low shore microhabitats, or because the species have found different pathways to deal with environmental stressors [58, 59]. Our findings show that within a relatively homogeneous environment, there are species-specific signals of selection, highlighting the importance of localized environmental and ecological forces potentially shaping species’ evolutionary trajectories. These findings promote using SNP datasets for conservation purposes to identify populations with heightened adaptive potential, even across relatively homogenous habitats, as these methods can elucidate areas with unique selective pressures with greater power than traditional markers [5, 7, 60].

### Pool-seq analyses and their implications for SNP calling and patterns of genomic variation

For each study species, the pattern in number of SNPs (identified by PoPoolation and PoPoolation2) generally follows that of the number of mapped reads (Tables 1 and 2), which suggests that the number of mapped reads per population influences the number of total SNPs per population. Further, the metrics calculated by PoPoolation all follow the same pattern as the number of SNPs and number of mapped reads (Table 1), indicating that population-specific parameters are potentially biased by the number of mapped reads and SNP coverage. However, the metrics calculated from all populations combined in Popoolation2 do not follow the same pattern as the number of mapped reads or number of SNPs (Table 2). Further, the allele frequency spectrum plots display less variation in allele frequencies for the Popoolation2 results (Additional file 1: Figure S1-S4), which also suggests that the strict resampling to even coverage across all populations in Popoolation2 led to less biased results. Therefore, the metrics derived from all populations combined are less likely to be influenced by methodological artifacts, and probably reflect actual biological processes. Given the uncertainty around potential biases associated with the population-specific calculations, the remainder of this article only refers to Popoolation2 results when discussing the genomic variation of the study species.

The number of SNPs per population varied both within and between species, and show noticeably more SNPs in the Granular limpet populations compared to the Cape urchin (average number of SNPs per population being ~ 52,000 and 6,700 were SNPs respectively; Table 2). This finding, in conjunction with higher levels of heterozygosity in the limpet populations (Table 2), is somewhat unexpected as there is ample evidence that urchin species harbor highly polymorphic individuals [61–63]. However, the interspecific difference in the number of SNPs is likely caused by the Granular limpet having a higher number of raw sequences, a longer average length and higher average quality of mapped reads [64]. For example, the average number of paired reads is 5.9 million for the limpet and 4.5 million for the urchin, plus the limpet samples have a total of 25 million mapped reads compared to the 19 million for the urchin (Table 1). Given that the number of raw sequences and mapped reads in turn affects the number of identified SNPs [64], it is difficult to compare SNP diversity between species.

Our results are of further interest, as the Cape urchin has a longer de novo reference sequence, and higher mean coverage than the Granular limpet, yet fewer total SNPs are recovered throughout the urchin populations in comparison (8,386 vs 55,409 SNPs). This result is most likely a consequence of the Cape urchin having more variation between populations than the Granular limpet. For example, the difference in the number of reads per population is 2.1 million reads for the urchin and 1.3 million reads for the limpet, whilst the difference in mean coverage is 149 and 59, respectively. As the total number of SNPs is calculated from all populations combined, if a SNP does not have sufficient coverage in at least one population, that SNP will be excluded from the overall count, which could explain the lower number of total SNPs in the urchin populations.

It should also be acknowledged that the patterns of genome-wide SNP variation may be influenced by ascertainment bias, which is when a selection of markers (usually those with high minor allele frequencies) affect inferences of the larger population, which is a problem experienced in many SNP analyses [65]. However, RAD-seq approaches are thought to have more unbiased population statistics due to higher number of sequenced genomic regions [66]. Furthermore, our large pool sizes and stringent SNP filtering protocols should also decrease the possible effects of ascertainment bias.

There is also the possibility that interspecific sequencing differences are influencing the de novo assemblies*.* One would expect the Cape urchin to have a larger de novo assembly, as the annotated genome for its respective taxonomic group (the Purple urchin, *S. purpuratus*, [67]), is 454 Mb larger than that of the Granular limpet (the Owl limpet, *L. gigantea*, [68]), yet our results show de novo assembly sizes to be similar between the two species. Molluscs are, in general, known to have a wider range of genome sizes than echinoderms, with sizes ranging from around 390 Mb to 5770 Mb, compared to 290 Mb to 4300 Mb [69]. The species in our study most likely show similar de novo assembly sizes due to the enzymatic activity of RAD-seq, which will result in similar sizes of raw reads, hence resulting in similar K values for the de novo assembly [70]. The DNA quantity (~ 29 and ~ 30 ng/μl) and quality (~ 32 and ~ 33 Phred scores) of the original pooled samples are also similar between species, which could have implications for de novo assembly sizes, yet a more in-depth analysis of the effects of quantity and quality of pooled samples on de novo assemblies is needed to address this theory.

While several studies suggest that Pool-seq provides accurate estimates of genomic variation [71–73], other studies express concerns about Pool-seq limitations and biases, and subsequently calls have been made for the standardization of a Pool-seq bioinformatics pipeline to increase the reliability of Pool-seq results [74, 75]. As Pool-seq is becoming more popular in genomic studies [76], it is important to understand the effects of differential amounts of genomic information per pool on diversity metrics, given the potential impacts applying these data in the management or conservation of natural resources.

### A comparative approach to identify areas of evolutionary uniqueness

The number of private SNPs across populations of both species show a non-geographical gradient (Table 2), suggesting that neither IBD, nor the regional oceanographic features, are likely driving the observed pattern. The IBD tests also show no significant isolation-by-distance from either the neutral or outlier datasets of either species (Additional file 1: Table S5). As the number of private SNPs is expected to be driven by gene flow and genetic drift rather than other evolutionary processes such as mutation and selection [77], we can assume that populations with high levels of private SNPs are demographically isolated to some degree.

Overall, the results from the private SNPs suggest that the northern populations, Port Nolloth and Hondeklipbaai, are evolutionarily unique with regards to the study species (Table 2). This finding could mean that these populations are experiencing environmental pressures either preventing SNPs from spreading to surrounding areas or selecting against SNPs from other populations. Another possible explanation for the uniqueness of this area is the occurrence of range expansions and associated population growth due to sea-level changes in the past 100,000 years, which might have facilitated previously isolated populations being re-integrated into the west coast meta-population [78]. Species distribution models based on paleoclimate temperature data of the Last Glacial Maximum (LGM; Seymour, Midgley & von der Heyden, pers. comm) suggest that west coast marine species shifted their ranges south, and that coastal marine species would have been locally extinct north of Jacobsbaai, with a subsequent range expansion northwards as sea levels and temperatures increased. Regardless of the processes that have shaped the array of private SNPs within the study species, our results indicate that the northern west coast of South Africa may possibly be a reservoir of genomic diversity for marine invertebrates not found elsewhere.

### Fine-scale phylogeographic patterns suggest complex evolutionary histories

The study species show different patterns of genomic variation and differentiation, providing further evidence that the evolutionary processes shaping marine biodiversity in South Africa are complex (Fig. 2; [79, 80]). For example, neither species shows a geographically ordered pattern in genomic differentiation (Fig. 2), which is observed for other marine invertebrates within the region [35, 37]. For both species, the genomic structuring differs between non-outlier loci and outlier loci (Fig. 2), which is expected as outlier loci are identified from their unique *F*_*ST*_ values. Increased genetic structuring in outlier loci has been discovered for other high gene flow marine species, most of which is attributed to historical population processes and local adaptation [14, 81–83]. However, outlier analyses are not without theoretical complications, often suffering from high rates of false positives [84]. In our case, the low levels of population structuring in our SNP datasets provide a less noisy neutral background for outliers to be detected from, making our outlier analyses more robust [85].

It is noteworthy that the genomic structuring of outlier loci for both species show Hondeklipbaai as being highly differentiated (Fig. 2), which suggests this finding is not due to chance alone. Of the Cape urchin populations, Port Nolloth and Hondeklipbaai are highly differentiated in outlier loci (Fig. 2), which is interesting as they are geographically close to one another. The high genomic distinctiveness of the northernmost populations could be due to local selection pressures acting on these populations [86], or from both species experiencing a recent expansion into this region, which would cause allele frequencies of all loci, including those selected on, to be different from the rest of the meta-population [87, 88]. At present, we can assume that the northern populations of both species have unique evolutionary histories, which makes them priority areas for the conservation of evolutionary processes.

With the K-means clustering analyses, we found no clear signal of population structure for both species, which contradicts previous structure analyses with mtDNA cytochrome oxidase I, where the Cape urchin displayed high levels of population structuring [35, 42]. Several environmental and biological features are probably shaping the shallow genetic structure of our study species, such as recent changes in demography or the strongly northward flowing Benguela Current and inshore eddie systems [89], although these are poorly understood for nearshore coastal environments [90]. Numerous phylogeographic studies have invoked life history traits as drivers of genetic structuring in marine species [83, 91, 92], however, our study species have notably different life history traits, with, PLD at roughly 50 days for the urchin and 10 days for the limpet [37]. There has been ample debate on life history traits as predictors of population structuring of marine invertebrate species, [93, 94], and hopefully a clearer picture will arise as more comparative genomic phylogeographic studies are conducted.

### Identifying functions of outlier loci

Even though there are generally low levels of genome-wide differentiation between populations for both species (Additional file 1: Tables S3 & S4), there are loci displaying significantly high levels of differentiation, classifying them as having a higher probability of being actively selected on. Of the outlier loci for each species, approximately 76% significantly match to the Owl limpet, *L. gigantea* genome and 57% significantly match that of the Purple urchin, *S. purpuratus*. Fewer significant BLAST results for the Cape urchin are most likely owing to the high levels of polymorphism in urchin genomes [61–63].

Between the two species, four contigs containing outlier loci had high probabilities of being related to histone proteins (Additional file 1: Tables S2). It is possible that the identification of histones as outlier loci could be a result of genetic hitchhiking [95], or due to histones being highly conserved, therefore making their identification far easier than rare or undescribed proteins. While histone variants are known to modify gene expression patterns within organisms [96, 97] few studies have investigated the influences of histone methylation on the adaptation of marine organisms [98], although histone loci have been proven to be diagnostic for sister species in recently diverged corals [99, 100]. Urchins are also known to have a large family of histone genes compared to other invertebrate species [101, 102]. Further, Zbawicka and co-authors [103] report four out of 20 outlier loci as histone genes within *Mytilus trossulus* and *Mytilus edulis* in the Baltic Sea. Ultimately, further investigation is needed to better understand the potential functional roles of histone variants within marine invertebrates to be able to state their adaptive significance within our study.

In addition to histone variants, both species displayed outlier-containing contigs matching to sequences within the Endonuclease/Exonuclease/Phosphatase family, and more specifically, to reverse transcriptases and mobile elements within this domain, which is not unusual as retrotransposons and retroelements are thought to be widespread throughout eukaryotic genomes [104, 105]. Retroelements are highly mobile genetic elements that are known to play significant roles in disease progression, stress reactions and embryogenesis, and are thought to be found in regions of the genome with reduced rates of recombination [106, 107]. Genes within these domains have been matched to outlier loci in previous genomic studies of other marine invertebrates [97, 108], however, the authors of these studies conclude that this finding is not a result of the annotated sequences not being under selection themselves, but rather linked to loci that are under putative selection.

The remaining contigs were matched to proteins of various domains and functions, including alpha tublins, ribonuclease H-like enzymes, homeodomain proteins, cadherins, and DNA breaking-rejoining enzymes. Most of these protein domains are either highly abundant throughout mollusc and echinoderm genomes and/or are highly conserved [109–111], and therefore are also likely not under selection themselves but again linked to genes under selection.

The only identified neighboring genes of the outlier loci were the pol-like protein of the *L. gigantea* genome and the cysteine-rich motor neuron 1 protein precursor of the *S. purpuratus* genome. The pol-like protein has been identified in outlier analyses of other marine invertebrates [112–114], and is expected to be involved with immunity and stress relief [115]. The cysteine-rich motor neuron 1 protein precursor is not commonly identified as a candidate gene, but has displayed differential expression in the sea cucumber *Apostichopus japonicas* [116]. The cysteine-rich motor neuron protein is predicted to assist with the development of the central nervous system by interacting with growth factors involved with motor neuron differentiation and survival [117]. Ultimately, further annotation of related genomes and more in-depth seascape genomic studies are required to further test the effects of environmental pressures on the Cape urchin and Granular limpet along the South African west coast.

### Comparative genomics: Local selective forces within a homogeneous environment

Notably, our study recovered different patterns from the spatial distribution of outlier loci, with ~ 24% shared by all limpet populations, but 50% shared by all urchin populations. Furthermore, the urchin populations have no private outlier loci, compared to the nine private outlier loci shown in the limpet samples. For the urchin, the high number of shared outlier SNPs could be caused by selection on standing genetic variation, high N_e_ or rather by the ‘transporter-hypothesis’, where gene flow spreads favorable adaptations between populations [118]. Our finding of high levels of shared outliers contradicts the results of Ravinet and co-authors [17] who found no shared outlier loci between three *L. saxatilis* populations within 10 km of each other. However, the authors attributed these results to either recent de novo mutations causing parallel evolution, unique selection pressures between sample locations generating non-shared outliers or complex polygenetic traits being responsible for similar phenotypes.

While most outlier loci are shared between at least two limpet populations, some outliers are private (Table 3), with Hondeklipbaai in particular having both the highest number of outlier and private outlier loci. Interestingly, the same population, Hondeklipbaai, has the lowest number of outlier and private outlier loci out of the urchin populations. This sample site has high levels of copper deposits in local sands [119], and copper exposure has been shown to be a strong selective agent within other marine organisms [120–122], but unfortunately, the lack of an annotated genome precludes a solid explanation.

It is likely that some of the differences in genomic variation reflect the unique ecological, biological and historical differences between the two study species. For example, although we sampled individuals from the same site, the species differ in their location on the rocky shore; the Granular limpet occurs higher on the shore where animals experience longer periods of emergence and hence more local environmental heterogeneity [123]. In contrast, the Cape urchin remains submerged in tidal pools, which might buffer factors such as rapid temperature changes and desiccation [123].

Our findings are not unexpected, as previous studies focusing on the adaptive traits of the periwinkle *Littorina saxatilis* [17, 25] found evidence of divergent selection between high and low shore ecotypes. In addition, Dong and Somero [124] compared the enzymatic activity of NADH dehydrogenase across six marine snail species within the *Lottia* genus and found that high shore species performed better at higher temperatures compared to those inhabiting the low-shore. Further, Galindo and co-authors [97] identified outliers associated with shell matrix, muscle and metabolic proteins (including NADH dehydrogenase) and reverse transcriptases from *L. saxatilis* individuals within either the high- or mid-shore ecotypes. Overall, it is likely that micro-environmental and ecological differences associated with variables such as temperature, exposure to wave action, competition and predation, play significant roles in shaping the outlier SNP patterns within the Granular limpet populations on the South African west coast.

As the Cape urchin is only found within the low-shore, and is known to protect itself from increases in air temperature and wave pressure by inhabiting sheltered rock pools [123], it is likely that the outlier SNPs found within this species are responding to environmental variables on a larger scale, such as CO_2_ and chlorophyll concentrations or SST, all of which have been identified as features shaping genetic diversity patterns in other urchin and marine species [125–128]. Furthermore, numerous studies have invoked SST as a strong determinant of driving genetic variation seascapes [11, 14, 81, 129]. In fact, several candidate genes putatively under selection are associated with changes in temperature in marine invertebrates, with many studies indicating that genes related to energy metabolism play important adaptive roles in changing temperature regimes [130–133]. We did not anticipate this to be a prominent selection force, as the South African west coast does not display a strong SST gradient [26], and accordingly to our predictions, no putatively selected loci associated with metabolic pathways were identified. It should be noted, however, that not all SNPs were matched to known genomic regions, which leaves uncertainty regarding which environmental or biological features are mostly responsible for the genomic patterns observed. Ultimately, it is most likely that there are additive or synergistic, rather than single, environmental and ecological processes shaping the evolutionary dynamics of marine taxa [134], including our study species.

## Conclusions

This is the first study to utilize a pooled RAD-seq approach to conduct comparative phylogeographic analyses, make inferences about population-based dynamics, and understand the evolutionary forces driving both intra- and inter-specific patterns of adaptive potential. It should be noted that this is a preliminary approach to properly identifying candidate genes for adaptation for conservation purposes, as the outlier loci and their functional roles still need to be confirmed and tested for both species. However, we can still make inferences about intraspecific population dynamics and adaptive potential with greater power with genome-wide SNP markers, which would not be possible with only a limited number of loci using traditional marker types. We detect signals of population differentiation and selection, suggesting that selective forces are acting on localized scales. Another interesting finding is that the two northernmost populations are genomically unique for both species, which is significant as it suggests that local environmental or ecological features are shaping the evolutionary trajectories of multiple coastal invertebrate species, even within this relatively environmentally homogeneous area. This preliminary finding provides a backdrop for a more in-depth seascape genomic analysis, which could help elucidate the possible environmental forces driving the genetic differentiation of marine invertebrates inhabiting these sites.

Our study also indicates that the Pool-seq methodology with de novo assemblies may be susceptible to differences in data quality. We argue that if Pool-seq is to be effective in comparing genetic diversity between non-model species, additional Pool-seq specific bioinformatic developments are required [47, 48, 135, 136]. We also found diverse patterns of selection between species, which supports the use of next generation sequencing techniques to carry out comparative phylogeography studies to assess the drivers of evolutionary processes of whole communities instead of single species.

Finally, we found evidence of differential selection among rocky shore sites, which suggests that environmental gradients within these microhabitats are also strong drivers of evolutionary change [18]. The complex patterns of private and outlier SNPs, both within and between species, suggests that studies aimed at identifying genomic variation with SNP datasets should not only focus on single species within predominantly heterogeneous environments, but also across different species and in seemingly homogeneous regions.

## Methods

### Study species and sampling protocol

The focal species were selected due to their high abundance within the high (Granular limpet; *S. granularis*) and low-mid (Cape urchin; *P. angulosus*) shores [39], as well as their highly differentiated genetic patterns within the study region (based on *COI*). Samples were collected from six rocky intertidal localities along the west coast of South Africa (Fig. 1) during the period of June to August 2015. Forty samples of both species were collected from each site and stored in 100% ethanol. The individuals from each of the six sample locations were labeled as separate ‘populations’.

Twenty-five micrograms of tissue were taken from each sample (gonad tissue from the urchin, foot tissue from the limpet). Genomic DNA was extracted using Qiagen DNeasy Blood & Tissue kit following the manufacturer’s protocols and extractions were then stored at -20 °C. At least 3 μg of high molecular weight DNA was measured into a concentration of 40 ng/μl using molecular grade H_2_0. For each species, all 40 individuals from each location were equimolarly pooled to create a total of 12 samples for Illumina sequencing. The pooled samples were flash frozen and sent to the Hawaii Institute of Marine Biology for library preparation [137] and v3 2 × 300 PE Mi-Seq Illumina sequencing through the Genetics Core Facility (GCF).

### DNA digestion and library preparation

Estimates of genetic variation are becoming more robust with the emergence of high-throughput sequencing methods, such as RAD-seq [138–141]. RAD-seq provides a relatively low-cost and efficient method to characterize SNPs over the entire genome, and is increasingly utilized to obtain genomic information from non-model organisms [142, 143]. Yet, because the cost of sequencing many individuals from multiple sites is often prohibitive, many studies apply a pooled sequencing (Pool-seq) approach, in which DNA from multiple individuals are combined and sequenced as a whole population [144, 145]. While Pool-seq does not allow individuals to be identified and compared within a population, it does allow for more individuals to be analyzed, which increases the power to estimate population-based allele frequencies [144, 146], and has been shown to be a viable approach to identify population genomic variation and detect local adaptation [145].

We employed a pooled ezRAD library preparation and sequencing approach [46], which uses a high-frequency restriction enzyme to fragment the DNA and obtain a reduced-representation sequencing library. For digestion and library preparation, we followed protocols described by Knapp and co-authors [137]. The size-selected DNA was prepared for sequencing using the KAPA Hyper Prep kit. Fragment size for the libraries was established using a bioanalyzer and quantified using qPCR as quality control measures before sequencing on the Illumina MiSeq platform.

### De novo assembly and data processing

The quality of raw reads from the MiSeq facility was first assessed with the FASTQC toolkit [147]. The reads were then trimmed with Trim Galore! (https://www.bioinformatics.babraham.ac.uk/projects/trim_galore/), trimming adapter and overrepresented sequences, as well as sections with bases having a Phred quality score lower than 20. As optimizing k-mer lengths for RAD sequences produces the highest quality assemblies [140] we conducted a de novo assembly with Spades v.3.5.0 [148] testing multiple k-mer lengths, and determined optimal k-mer lengths of 81 for the Cape urchin and 91 for the Granular limpet. Assembly statistics, such as assembly length, longest contig, and N50 and L50 lengths were calculated with QUAST v4.1.1 [149].

As semi-global alignment and realignment of unmapped reads is recommended for pooled samples [140], we used BWA-MEM [150], following the same parameters as in Toonen et al. [46], to map the filtered reads onto the de novo reference sequences. Mapping results (number of mapped versus unmapped reads) were calculated using the ‘stats.idx’ command in SAMtools v.1.3 [151]. The resulting SAM files were converted to BAM files with SAMtools, undergoing further filtering to discard all reads not mapped in a proper pair, reads not in a primary alignment and reads with a mapping quality score under 20. The BAM files were sorted and indexed, and then used to call variants with the ‘mpileup’ command in SAMtools, using a minimum quality score of 20 and maximum depth of 1000 reads per locus.

### Estimating population genomic variation

The population-specific number of SNPs, nucleotide diversity (Tajima’s *π*) and population mutation rate (Watterson’s *θ*_*W*_), were calculated for each population using the ‘variance-sliding’ command in PoPoolation [47]. The population specific number of SNPs, *π*, and *θ*_*W*_ were all calculated using a sliding window of 1,000 base pairs (bp), which was chosen after testing windows of 100, 500 and 1,000 bp. To standardize for sequencing biases, we subsampled for uniform coverage (minimum coverage of 10 and maximum coverage of 200) and set a minimum count (i.e. the number of times the allele appears) of four and a quality score of 20.

As the above-mentioned metrics were all calculated from pileup files containing only SNPs found within each individual population, we also wanted to calculate the number of SNPs per population from all samples combined. To do this, the mpileup file was converted in PoPoolation2 [48], producing a sync file indicating allele counts across all populations. The number of total SNPs and number of private SNPs were then identified from the allele counts given by the ‘snp-frequency-diff’ command in PoPoolation2. As these metrics are derived from all populations combined, to account for the increase in reads we increased the minimum count to eight, the minimum coverage to 40, and the maximum coverage to 500 to call SNPs at this stage. As Popoolation2 is not capable of calculating diversity indices such as Tajima’s *π* and Watterson’s *θ*_*W*_, total heterozygosity was calculated from the GenePop file used to detect outliers (see section below) using Genodive [152].

To assess the effects of population-specific resampling on intraspecific variation, we compared the frequencies of SNPs calculated by both Popoolation and Popoolation2. As allele frequencies are not available for Pool-seq, we used the allele count over allele coverage to represent allele frequencies.

### Detection of selection footprints

Given the uncertainty around RAD-seq, Pool-seq, and outlier detection methods in general [153–155], we followed the current trend in the literature and applied multiple outlier analyses to detect potential outlier loci with increased stringency. To identify outlier loci, we first converted the sync files created in PoPoolation2 to GenePop files, using the PoPoolation2 command ‘subsample_sync2GenePop’. This step underwent the same filtering as the ‘snp-frequency-diff’ command, with a minimum allele count of eight, minimum coverage of 40, maximum coverage of 500, as well as a target coverage of 40 to best simulate a GenePop file from 40 individually genotyped individuals. GenePop files were further edited using custom Perl script, to merge all contigs and identify locus positions.

The edited GenePop files were converted into Bayescan files using PGDSpider2 v2.1.03 [156]. The first outlier detection method, Bayescan v.2.1 [157], was run with 20 pilot runs, 10,000 iterations and a burn-in of 50,000, and 55,000 reversible-jump MCMC chains, using a prior odds value of 1,000, a thinning interval of 10 and a false discovery rate (FDR) of 0.05. The second approach, PCAdapt, was used to detect outlier loci based on principal component analyses [49]. Input files were created from the allele counts file produced by the ‘snp-frequency-diff’ command in PoPoolation2. Six principal components were used for both species, and outliers were identified as loci with a q-value lower or equal to 0.05.

To evaluate the functional roles of the outlier loci that were chosen by both methods, the contigs associated with each outlier locus were subject to BLASTX searches, using the non-redundant protein sequences database and an E-value cut off of 10^− 5^ [158]. To identify neighboring genes of the outlier loci, we aligned the outlier contigs onto the annotated genomes of *L. gigantea* and *S. purpuratus,* then BLASTed the flanking regions within 2 KB of either side of the contig, using the same mapping and search parameters listed above.

### Estimating genomic population structuring

Genetic population structure was characterized by pairwise *F*_ST_ values calculated by the ‘sliding-fst’ command in PoPoolation2, using a minimum count of eight and coverage of 40 and a maximum coverage of 500. To visualize the genomic differentiation between the populations for each species, we created Principal Coordinates Analysis (PCoA) plots based on the average pairwise *F*_ST_ values for both the putatively neutral (excluding outlier) and selected (outlier) SNPs using the vegan package in R-studio [159]. To evaluate genetic structure, we ran K-means clustering analyses separately on the neutral and outlier datasets with fastStructure v1 [160]. For each dataset, we tested K values from one to ten, with the prior parameter set to both ‘simple’, with the seed parameter set to 100. To test for isolation-by-distance, we performed Mantel tests with log-transformed geographic distances between sample locations and linearized *F*_*ST*_ values [*F*_*ST*_ / (1-*F*_*ST*_)], using the vegan package in R. We ran three types of Mantel tests (Pearson, Spearman, and Kendall) on the full, and putatively neutral, and selected datasets for each species.

## Additional file

Additional file 1: **Tables S1** – **S5** and **Figure S1** – **S4** as referred to in the text. (DOCX 1086 kb)

## Abbreviations

COI: Cytochrome oxidase I
IBD: Isolation by distance
LGM: Last glacial maximum
mtDNA: Mitochondrial DNA
N_e_: Effective population size
PLD: Pelagic larval duration
RAD: Restriction site associated DNA
SNP: Single nucleotide polymorphism
SST: Sea surface temperature
