# Supplementary material for: Complex signatures of genomic variation of two non-model marine species in a homogeneous environment
Source: BMC Genomics. 2018 May 9;19:347. doi: 10.1186/s12864-018-4721-y (PMC5944137; doi:10.1186/s12864-018-4721-y)
Supplement: Supplementary file 1 — Tables S1 – S5 and Figure S1 – S4 as referred to in the text. (DOCX 1086 kb) [file 12864_2018_4721_MOESM1_ESM.docx]

**Supplemental Information for:**

Complex signatures of genomic variation of two non-model marine species in a homogeneous environment

Erica S. Nielsen, Romina Henriques, Robert J. Toonen, Ingrid Knapp, Baocheng Guo, Sophie von der Heyden

**Table of Contents:**

| Table S1 | Page 2 |
| --- | --- |
| Figure S1  Figure S2  Figure S3  Figure S4  Table S2  Tables S3-S5 | Page 3  Page 3  Page 4  Page 4  Page 5  Page 6 |
|  |  |

Table S1- Assembly statistics, such as number of contigs at certain lengths, total assembly length, N50 and L50 lengths, and number of unknown bases per kilobase (# N's per 100 kbp) are reported for both the limpet, *S. granularis*, and the urchin, *P. angulosus*.

| Assembly statistics | *S. granularis* | *P. angulosus* |
| --- | --- | --- |
| # contigs (>= 0 bp) | 452,948 | 453,847 |
| # contigs (>= 1000 bp) | 31,780 | 35,552 |
| # contigs (>= 5000 bp) | 112 | 291 |
| # contigs (>= 10000 bp) | 2 | 15 |
| # contigs (>= 25000 bp) | 0 | 0 |
| # contigs (>= 50000 bp) | 0 | 0 |
| Total length (>= 0 bp) | 268,506,318 | 284,276,390 |
| Total length (>= 1000 bp) | 43,680,585 | 51,161,817 |
| Total length (>= 5000 bp) | 718,607 | 1,956,681 |
| Total length (>= 10000 bp) | 23,712 | 187,656 |
| Total length (>= 25000 bp) | 0 | 0 |
| Total length (>= 50000 bp) | 0 | 0 |
| # contigs_500 | 242,030 | 265,371 |
| Largest contig | 12,107 | 16,348 |
| Total length | 180,275,903 | 199,842,857 |
| GC (%) | 37.14 | 36.35 |
| N50 | 717 | 719 |
| N75 | 583 | 581 |
| L50 | 87,790 | 94,187 |
| L75 | 158,120 | 172,157 |
| # N's per 100 kbp | 0 | 0.01 |

Figure S1 – The allele frequency spectrum plots based on Popoolation SNPs for the following *P. angulosus* populations: A) Sea Point, B) Jacobsbaai, C) Lambertsbaai, D) Brandsebaai, E) Hondeklipbaai, F) Port Nolloth.

Figure S2 - The allele frequency spectrum plots based on Popoolation2 SNPs for the following *P. angulosus* populations: A) Sea Point, B) Jacobsbaai, C) Lambertsbaai, D) Brandsebaai, E) Hondeklipbaai, F) Port Nolloth.

Figure S3 - The allele frequency spectrum plots based on Popoolation SNPs for the following *S. granularis* populations: A) Sea Point, B) Jacobsbaai, C) Lambertsbaai, D) Brandsebaai, E) Hondeklipbaai, F) Port Nolloth.

Figure S4 - The allele frequency spectrum plots based on Popoolation2 SNPs for the following *P. angulosus* populations: A) Sea Point, B) Jacobsbaai, C) Lambertsbaai, D) Brandsebaai, E) Hondeklipbaai, F) Port Nolloth.

Table S2 – The BLASTX results for both species are shown with the identified protein domains and their relevant E-values. The contig or queary lengths are also given with the percentage of that length that matches the identified protein (% identical).

| Query length | | | Protein Domain name | | Query cover | E-values | % identical |
| --- | --- | --- | --- | --- | --- | --- | --- |
| *S. granularis* | | | | | | | |
| 11308 | | | | Histone H2B | 20% | 1,00E-54 | 100% |
| 1258 | | | | Histone 2A | 21% | 6,00E-40 | 100% |
| 1595 | | | | Homeodomain | 35% | 2,00E-51 | 49% |
| 178933 | | | | Alpha tublin/Beta/Alpha domain interface | 85% | 5,00E-117 | 98% |
| 203 | | | | Exonuclease-Endonuclease-Phoshatase (EEP) domain superfamily | 29% | 4,00E-88 | 41% |
| 3402 | | | | Ribonuclease H-like superfamily | 24% | 6,00E-11 | 32% |
| 802 | | | | Histone H4 | 9% | 1,00E-47 | 100% |
| *P. angulosus* | | | | | | | |
| 1155 | | Histone H3 | | | 14% | 9,00E-88 | 100% |
| 552 | | Endonuclease/Exonuclease/Phosphatase family | | | 43% | 1,00E-174 | 61% |
| 56 | | RNA-directed DNA polymerase from mobile element jockey-like | | | 39% | 3,00E-89 | 26% |
| 85 | | DIRS1 family of RNase HI in long-term repeat retroelements | | | 35% | 1,00E-133 | 62% |
|  | | | | | | |  |

Table S3 - Average pairwise *F*_ST_ calculated by Popoolation2 among the six sample sites for the *S. granulari*s.

|  | SP | JB | LB | BB | HB | PN |
| --- | --- | --- | --- | --- | --- | --- |
| SP |  |  |  |  |  |  |
| JB | 0.010 |  |  |  |  |  |
| LB | 0.010 | 0.009 |  |  |  |  |
| BB | 0.009 | 0.008 | 0.008 |  |  |  |
| HB | 0.010 | 0.010 | 0.010 | 0.009 |  |  |
| PN | 0.013 | 0.013 | 0.012 | 0.012 | 0.013 |  |
|  |  |  |  |  |  |  |

Table S4 - Average pairwise *F*_ST_ calculated by Popoolation2 among the six sample sites for *P. angulosus*.

|  | SP | JB | LB | BB | HB | PN |
| --- | --- | --- | --- | --- | --- | --- |
| SP |  |  |  |  |  |  |
| JB | 0.014 |  |  |  |  |  |
| LB | 0.013 | 0.008 |  |  |  |  |
| BB | 0.013 | 0.007 | 0.006 |  |  |  |
| HB | 0.014 | 0.014 | 0.014 | 0.015 |  |  |
| PN | 0.015 | 0.013 | 0.011 | 0.010 | 0.019 |  |

Table S5 – Results from isolation by distance (IBD) tests on all SNPs, excluding outlier SNPs, and solely outlier SNPs. The results are provided for Mantel tests using Pearson, Spearman, and Kendall correlations.

|  | All SNPs | | Non-outlier SNPs | | Outlier SNPs | |
| --- | --- | --- | --- | --- | --- | --- |
|  | R | P-value | R | P-value | R | P-value |
| *S. granularis* | | | | | | |
| Pearson | -0.11 | 0.62 | 0.20 | 0.24 | -0.14 | 0.69 |
| Spearman | -0.15 | 0.70 | 0.17 | 0.27 | 0.18 | 0.29 |
| Kendall | 0.07 | 0.65 | 0.14 | 0.26 | 0.12 | 0.32 |
| *P. anglulosus* | | | | | | |
| Pearson | -0.27 | 0.87 | 0.44 | 0.04 | 0.16 | 0.34 |
| Spearman | -0.20 | 0.75 | 0.39 | 0.11 | 0.30 | 0.16 |
| Kendall | -0.14 | 0.78 | 0.30 | 0.11 | 0.24 | 0.14 |
